# TRBC1/TRBC2 Immunophenotyping Provides Added Value During the Diagnostic Workup of T‐Cell Lymphoma

**DOI:** 10.1002/jha2.70192

**Published:** 2025-12-22

**Authors:** Joseph Taylor, Tasneem Lakum, Shaun Bevan, Tanya Freeman, Jesca Boot, Carola Maria Bigogno, Juliet Raine, Edward Hookway, Hanine Medani, Maria Calaminici, Jessica Okosun, Marianne Grantham, Shamzah Araf, Matthew Smith, Tom Butler, Timothy Farren

**Affiliations:** ^1^ Department of Clinical Haematology Royal London Hospital Barts Health NHS Trust London UK; ^2^ Precision Health University Research Institute Queen Mary University of London London UK; ^3^ Specialist Integrated Haematological Malignancy Diagnostic Service Royal London Hospital Barts Health NHS Trust London UK; ^4^ National Acute Lymphoblastic Leukaemia MRD Service Barts Health NHS Trust London UK; ^5^ Department of Haemato‐Oncology St Bartholomew's Hospital Barts Health NHS Trust London UK; ^6^ Barts Cancer Institute Queen Mary University of London London UK; ^7^ Department of Cellular Pathology Royal London Hospital Barts Health NHS Trust London UK

**Keywords:** clonality, diagnosis, immunophenotyping, T cell, T‐cell lymphoma

## Abstract

**Background:**

Molecular T‐cell receptor (TCR) clonality testing cannot link clonality to immunophenotype, limiting discrimination between malignant and clonal reactive T‐cell expansions.

**Methods:**

We evaluated TRBC1/TRBC2 immunophenotyping in 50 consecutive cases with suspected T‐cell malignancy, assessing restriction (ratio > 10) across T‐cell subsets.

**Results:**

TRBC immunophenotyping showed 100% sensitivity for T‐cell malignancy if assessed within subsets or phenotypically abnormal populations. Malignant clones predominantly showed CD4αβ/CD8αβ restriction (18/21), while reactive clones predominantly showed γδ/dual‐positive/dual‐negative restriction (15/16), enabling enhanced discrimination.

**Conclusion:**

TRBC immunophenotyping provides sensitive clonality detection with phenotypic lineage assignment, adding diagnostic clarity beyond molecular testing and supporting integration into routine workflows.

**Trial Registration**: The authors have confirmed clinical trial registration is not needed for this submission.

1

T‐cell lymphomas are diagnostically challenging due to the difficulty in distinguishing malignant disorders from expansions of reactive T‐cell clones [1]. We report the clinical evaluation of T‐cell receptor beta chain (TRBC) immunophenotyping within a Specialist Integrated Haematology Malignancy Diagnostics (SIHMDS) Centre, evaluating performance against the current standard of molecular T‐cell receptor (TCR) clonality testing. We demonstrate that integrating TRBC immunophenotyping with molecular testing adds diagnostic clarity in an area where diagnosis can be protracted and challenging. We show that immunophenotypic assignment of lineage to clonality enhances discrimination between malignant and reactive clones, due to enrichment for αβ T‐cell subsets in malignancy and atypical phenotypes (γδ, dual‐positive [DP] and dual‐negative [DN]) in reactive expansions. We further demonstrate that lineage assignment by molecular TCR β or γ rearrangement is not reliable, as genomic rearrangements do not consistently correlate with receptor expression.

In B‐cell malignancy, assessment of kappa or lambda light‐chain restriction is well‐established for the detection of a dominant clone [2, 3, 4]. As such, molecular B‐cell clonality testing is unnecessary where light‐chain restriction by immunophenotyping is demonstrable. In T‐cell malignancy, diagnostic practice relies on molecular TCR clonality testing [2]. TCR clonality tests assess β and γ TCR rearrangement, identifying clonality in most but not all T‐cell malignancies from a pooled T‐cell population [5]. Molecular testing is highly sensitive but hampered by limited specificity due to the detection of dominant sub‐clones among reactive T‐cell expansions, which are common both in non‐malignant settings and other malignancies [6, 7]. There is no direct method to link a molecular clonality result and an immunophenotypically abnormal clone, leaving a degree of uncertainty as to whether the phenotyped population corresponds to the clonal one. This leads to ambiguity and highlights an unmet need for more precise diagnostic tools in the evaluation of suspected T‐cell malignancies.

Until recently, flow‐cytometric assessment of clonality relied upon assessment of 24 different TCRβ‐variable families, a time‐ and resource‐intensive process [8, 9]. However, novel antibodies targeting TCRβ‐constant regions now permit the division of T cells into two broad beta chain families (TRBC1 and TRBC2) [10, 11, 12]. This permits rapid assessment of T‐cell restriction, and recent studies have demonstrated utility in research and diagnostic settings, but widespread adoption and validation in routine clinical diagnostics remain limited [11, 13, 14]. This approach allows the assignment of clonality to a specific phenotype, thereby addressing a key limitation of molecular testing.

We assessed TRBC restriction by immunophenotyping to supplement routine diagnostic practice. TRBC1 and TRBC2 antibodies (Beckman Coulter, cytoplasmic) were incorporated in a panel alongside CD3, CD4, CD5, CD8, CD10, CD19, CD38, CD45, CD56, CD57 and TCRαβ (BD Biosciences). Intracellular staining utilised IntraStain from Dako/Agilent. Samples were processed on BD FACSLyric analysers. Comparison was made to β/γ TCR clonality testing. Cases were selected sequentially from cases referred to the SIHMDS for possible blood cancer. Samples were analysed with routine screening panels assessing T, B, NK and myeloid components. Samples were included in the study on identification of a T‐cell expansion during screening or when clinical suspicion of T‐cell malignancy was specified by the referrer. A total of 50 cases were analysed. On completion of investigations, 20 received a formal diagnosis of T‐cell non‐Hodgkin lymphoma (T‐NHL), 1 T‐lymphoblastic leukaemia/lymphoma, 1 lymphocyte‐variant hypereosinophilic syndrome (L‐HES), 1 T‐cell clone of uncertain significance (T‐CUS) and 27 were classified as reactive after review of diagnostic material and clinical notes by an SIHMDS Consultant. A T‐cell population was considered clonal when the TRBC1:TRBC2 or TRBC2:TRBC1 ratio was greater than 10 [11]. Clonality was assessed in the global CD3+ population, then reassessed on gating to the CD4+, CD8+, CD4+CD8+ (DP) and CD4–CD8− (DN) subsets and categorising by αβ and γδ status. Further assessment was made in specific, identified subsets with abnormal phenotype.

TRBC immunophenotyping prospectively identified 21 of 22 cases ultimately diagnosed with T‐cell malignancy, L‐HES or T‐CUS. In one case, loss of TCR expression rendered assessment technically unfeasible. Importantly, assessment of clonality gating on the total CD3+ population alone lacked sufficient sensitivity for diagnosis (Figure 1b). In six cases of T‐cell malignancy, TRBC ratios fell below the diagnostic threshold (< 10) within the total CD3+ population, but exceeded the threshold (> 10) when gated subsets were analysed: CD4+, CD8+ (Figure 1c), DN or DP compartments (not shown). In two additional cases, one CD4αβ+ adult T‐cell leukaemia/lymphoma (ATLL) and one CD4αβ+ peripheral T‐cell lymphoma not otherwise specified (PTCL NOS), TRBC ratios remained below the threshold (< 10) even within CD8αβ/CD4αβ compartments, but clonality was demonstrated on gating to phenotypically aberrant subpopulations (Figure S1). Non‐malignant clonal expansions were seen in 16 cases with TRBC ratios greater than 10. These generally occurred at lower ratios than in malignant T‐cell disorders; however, it was not possible to establish a threshold to robustly discriminate between clonality in reactive expansions and malignant disorders. Overall, TRBC immunophenotyping showed 100% sensitivity and negative predictive value for the detection of T‐cell malignancy, L‐HES or T‐CUS, but detection of non‐malignant clonal T‐cell expansions meant that specificity and positive predictive value were only 48% and 61%, respectively (Table S1).

**FIGURE 1:**
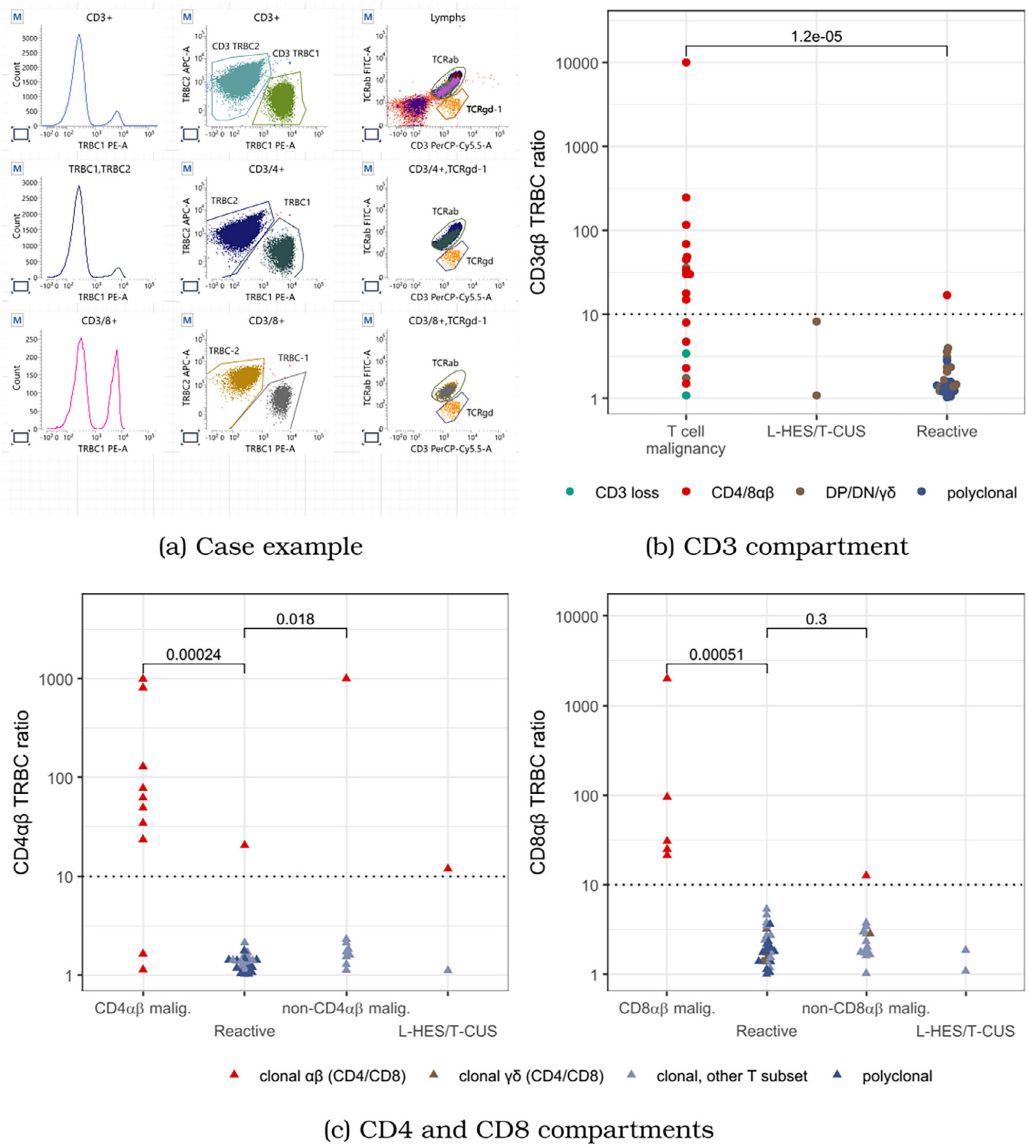
T‐cell receptor beta chain ratios in T‐cell malignancy and reactive conditions. (a) Case example of clonality detected within CD3 and CD4 compartments and identified as TRBC2 restriction within the CD4αβ+ compartment. (b) TRBC ratio within the global CD3+ compartment. Colours represent the T‐cell compartment where clonality was identified as originating from after gating to sub‐populations. Multiple cases with a final diagnosis of T‐cell malignancy are not identified by a TRBC ratio of 10 applied to the global CD3+ compartment. (c) TRBC ratios within CD4αβ (left) and CD8αβ (right) gates. Colours represent αβ or γδ clonality within the CD4 or CD8 compartment, respectively. Points designated non‐CD4αβ/CD8αβ malignancy represent cases where a diagnosis of T‐cell malignancy was made based upon clonality seen in a different compartment. Isolated cases of CD4αβ malignancy with TRBC ratio < 10 were diagnosed on gating to phenotypically abnormal subpopulations (See Figure S1).

Concordance between TRBC immunophenotyping and molecular TCR analysis was high but not complete (Figure S2). Twenty‐three out of 26 cases where both techniques were performed showed concordant results. In each of three cases with discrepant results, a final diagnosis of reactive T‐cell proliferation was made. In an additional 19 cases, molecular TCR testing failed (five cases) or was not performed.

Despite similar sensitivity and specificity to molecular testing, TRBC immunophenotyping provided additional information, assisting in the discrimination between malignant and clonal expansion through the ability to delineate the phenotype of the clonal population.

In T‐cell malignancy, TRBC immunophenotyping provided confirmation of clonality within the phenotypic subset expected for the specific diagnostic entity. For example, clonality within the CD8αβ subset for a diagnosis of T large granulocytic leukaemia (T‐LGL). Overall, clonality within a CD4αβ or CD8αβ population was highly suggestive of T‐cell malignancy, consistent with the fact that most T‐cell malignancies are of CD4αβ or CD8αβ origin. Conversely, clonality within atypical T‐cell lineages was uncommon in the setting of malignancy, accounting for 3 of 21 cases (Figure 2a). These cases were seen in malignancies associated with atypical T‐cell subsets: γδ clonality in hepatosplenic T‐cell lymphoma (HSTCL), DN clonality in ENKTL and DP clonality in T‐PLL, all well‐described phenotypes for their respective malignancies.

**FIGURE 2:**
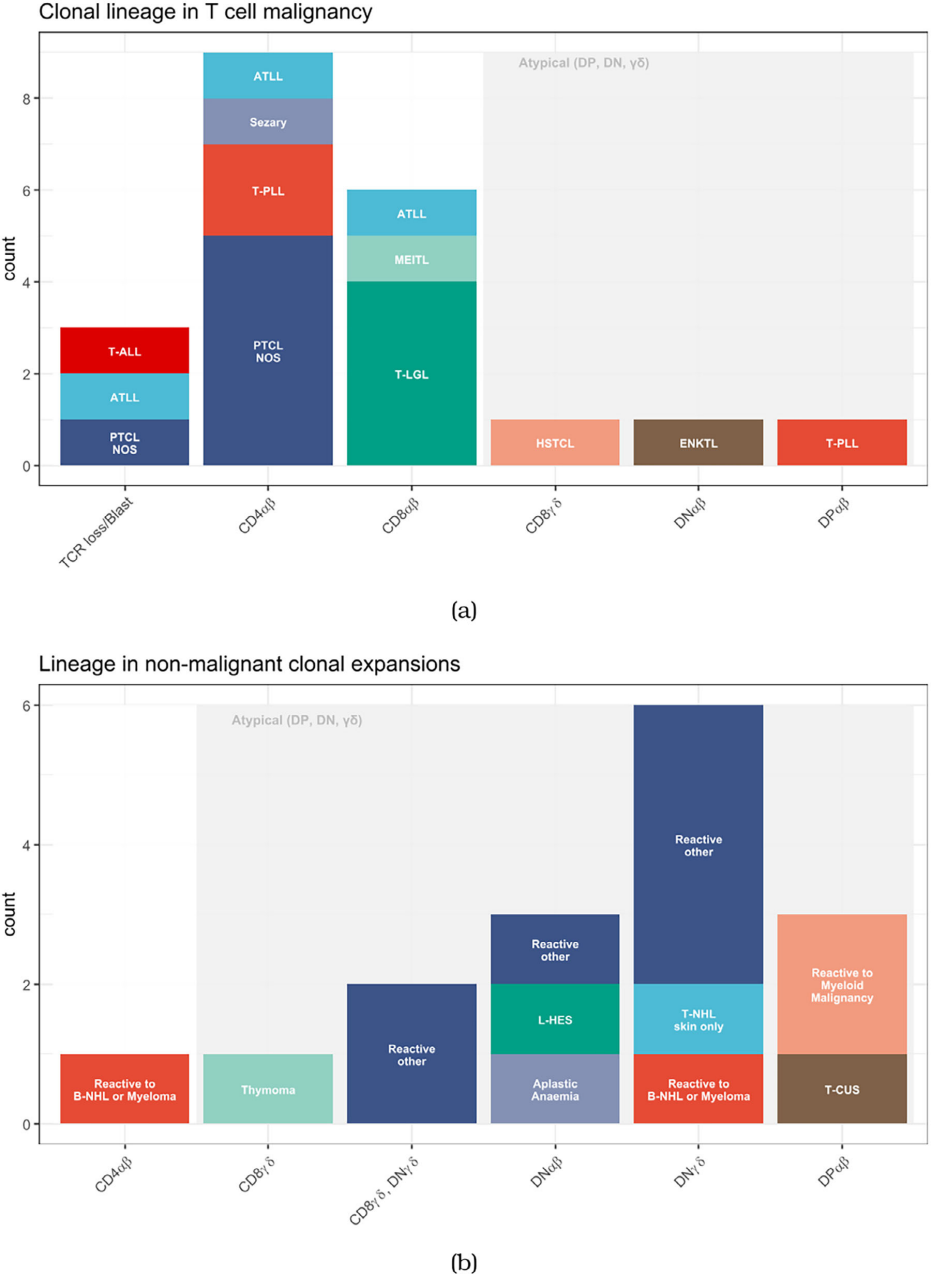
Investigation of lineage of malignant and reactive T‐cell clones. (a) T‐cell clones in malignancy are frequently of conventional (CD4αβ or CD8αβ) lineage. Atypical (dual‐positive, dual‐negative and γδ) lineages are uncommon. (b) T‐cell clones in reactive expansions are predominantly of atypical lineage, with only rare examples of conventional lineage.

In contrast to the pattern in malignancy, clonality within atypical lineages (γδ, DP or DN) was common in reactive T‐cell expansions. Only one instance of a CD4αβ clone was identified in the reactive setting, seen in the context of follicular lymphoma. Fifteen of sixteen non‐malignant clonal expansions were γδ, DP or DN (Figure 2b). One case highlighted the added diagnostic value of TRBC immunophenotyping: A patient with a history of renal transplantation presented with neutropenia. Immunophenotyping revealed both a T‐large granular lymphocyte population and a DN T‐cell expansion. Molecular TCR analyses reported a clonal population. Bone marrow histology was inconclusive, and *STAT3*/*STAT5B* mutations (frequently mutated in T‐LGL) were not detected. TRBC immunophenotyping demonstrated that the clonal population detected by molecular TCR analysis corresponded to the DNγδ T‐cell expansion, not the T‐large granular lymphocyte population, effectively ruling out T‐LGL leukaemia. Correlation of clonality with the expected malignant phenotype was therefore valuable in reinforcing diagnostic accuracy.

The order of *TCR* gene recombination provides information as to the lineage of a clonal expansion [15]. During T‐cell development, *TCRG* and *TCRD*, alongside incomplete *TCRB* rearrangement, occur first, followed by the completion of *TCRB* and *TCRA* rearrangement [15, 16]. Therefore, αβ and γδ lineages can theoretically be distinguished because γδ malignancies exhibit γ rearrangement, while αβ exhibit β and γ [15]. However, we find poor concordance between gene rearrangement and phenotype. This may be due to unexpected and uncontrolled mutation occurring in the context of malignancy. Consistent with this, we have observed β and γ *TCR* gene rearrangements in B‐lymphoblastic leukaemia where no rearrangement would be expected. This highlights that molecular profiles do not reliably correspond to TCR expression and that immunophenotyping is required to robustly identify lineage.

In summary, we report validation of TRBC antibodies in routine diagnostic care within a diagnostic referral centre. Our findings demonstrate that TRBC immunophenotyping enables rapid screening for T‐cell malignancy with low failure rate. Our data support that a TRBC ratio cut‐off of > 10 effectively identifies T‐cell malignancies after gating to T‐cell subtypes or populations with abnormal phenotype, but that rigid application of this threshold to the global CD3+ population and even occasionally within subsets was insufficient for reliable detection of a clonal population. TRBC immunophenotyping, like molecular testing, it was highly sensitive but suffered from low specificity due to the detection of reactive T‐cell clones. However, our data highlights that pairing immunophenotyping with clonality adds diagnostic value both in the confirmation and exclusion of T‐cell malignancy. We further highlight that while molecular methods theoretically allow similar assignment of αβ or γδ lineage, this does not consistently correlate with TCR expression.

Interpreted in the context of clinical findings and multi‐modal diagnostics, TRBC immunophenotyping provides rapid, reliable clonality assessment and critical discriminative power beyond a positive TCR clonality result: CD4αβ or CD8αβ restricted clones support a diagnosis of T‐cell lymphoma versus γδ, DP or DN clones more frequently seen in reactive settings. The addition of TRBC immunophenotyping into routine diagnostic workflows, therefore, offers a valuable tool to combat the specificity gap with conventional clonality testing while maintaining sensitivity and improving diagnostic clarity in a challenging disease spectrum.

## Author Contributions

J.T. wrote the manuscript. J.T., T.L., S.B., M.G., and T.F. performed analysis and data curation. T.L., S.B., T.F., J.B., C.M.B., J.R., E.H., H.M., M.C., J.O., S.A., M.G., M.S., T.B., and T.F. reviewed and edited. T.F. conceived and funded the study.

## Funding

This study was funded by Barts Health NHS Trust as part of internal clinical validation.

## Ethics Statement

The authors have nothing to report.

## Conflicts of Interest

The authors declare no conflicts of interest.

## Supporting information



Supporting Information

## Data Availability

Anonymised data available on request from the corresponding author.
