# Supplementary material for: TRBC1/TRBC2 Immunophenotyping Provides Added Value During the Diagnostic Workup of T‐Cell Lymphoma
Source: EJHaem. 2025 Dec 22;6(6):e70192. doi: 10.1002/jha2.70192 (PMC12721119; doi:10.1002/jha2.70192)
Supplement: Supplementary file 1 — Supporting Information [file JHA2-6-e70192-s001.pdf]

Supplementary information.

Table 1

Sensitivity / Specificity Table			
	Final Diagnosis		
TRBC Clonality	T-NHL, L-HES or T-CUS	Reactive	Total
Positive	22	14	36
Negative	0	13	13
Total	22	27	
Sensitivity = 1, Specificity = 0.48<br>Positive Predictive Value = 0.61, Negative Predictive Value = 1			

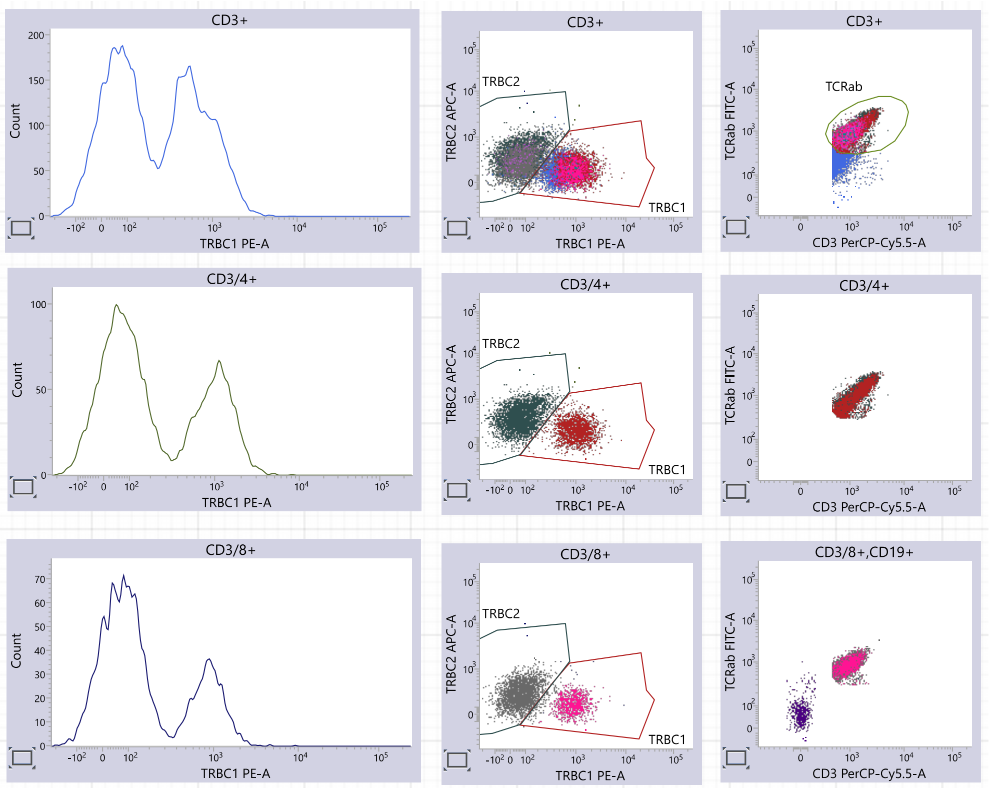

(a) Polyclonal CD3, CD4 and CD8 compartments

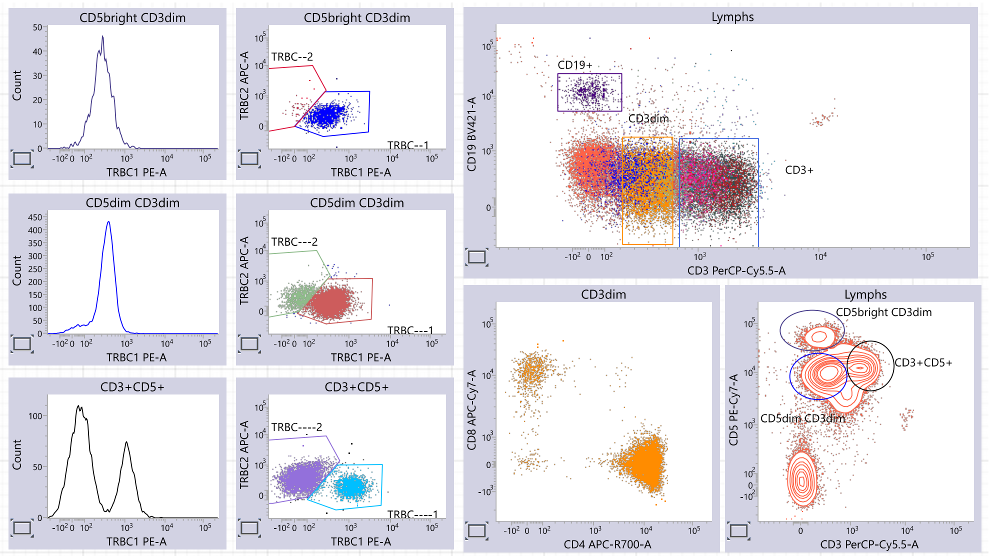

(b) Clonality within CD4+ phenotypically abnormal subpopulation

Supplementary Figure 1: **Identification of clonality within a subpopulation of abnormal phenotype** **a** Assessment of global CD3, CD4 and CD8 subsets show balanced TRBC1 and TRBC2 expression consistent with polyclonal T-cells. **b** Gating to phenotypically abnormal CD4+CD3weak population confirms clonality. CD4+ ATLL with concordant phenotype is subsequently confirmed on lymph node biopsy.

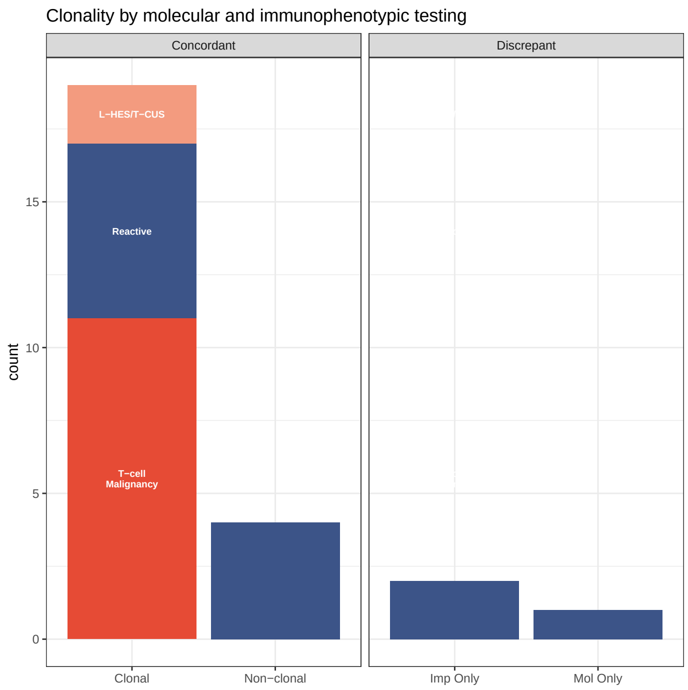

(a)

Supplementary Figure 2: Comparison of concordance of clonality assignment by molecular and immunophenotyping. High but incomplete concordance between molecular and immunophenotyping methods (88 %). 3 cases with lack of concordance between techniques.
